# ‘*Generalistas’*: the GPs of Madrid

**DOI:** 10.1080/17571472.2016.1225630

**Published:** 2016-09-03

**Authors:** Hajra Siraj

**Affiliations:** ^a^ The Riverside Vocational Training Scheme, Emperors’ Gate Surgery, Kensington, UK

The *Hippokrates* exchange was an eye-opening experience for me, enabling me to get a unique insight of primary care in another country.^1^ It really broadened my experiences, and taught me practical and new ideas to bring home. Colleagues I have spoken to about the scheme since returning have been inspired to participate. I hope my article will increase awareness and encourage others to get involved with this fantastic program. Despite the similarities between the healthcare systems of Spain and the UK, a number of subtle yet significant distinctions exist, with the power to alter one’s perspective on the realities of life as a GP.

As a final-year GP trainee, I was fortunate enough to experience life as a Spanish GP through the Hippokrates Exchange Program. This allows trainees, and GPs within five years of qualification, to shadow a family doctor in one of twenty-eight European countries. I spent two weeks in Madrid shadowing a seasoned GP named Dr Francisco Camarelles.

My placement was at the ‘Infanta Mercedes’ health centre, overlooking the world famous San Bernabeu football stadium. It is a large clinic of eighteen GPs, each paired with a nurse, seven trainees, two paediatricians, and ten administrative staff. The patient population is a diverse group of thirty-two thousand, and I saw patients from Camarelles’ list of two thousand.

Unlike the UK, the GPs here are only allocated seven minutes per appointment. Patients can also ‘walk in’ if their problem is urgent, which means that – on average – a staggering forty patients are seen in a single day.

These GPs also have no allocated time for paperwork; it’s all done during the consultation. Patients bring in copies of their discharge summaries, clinic letters and results, which are then interpreted and recorded electronically. Likewise, referral letters are written then and there. I liked the way this system hands over some responsibility to the patient; by managing their own healthcare, it helps to bridge the gap between primary and secondary sectors.

But though the working hours here are fewer (with doctors either working 8am–3pm or 2pm–9pm) compared to the UK, there is little flexibility in the schedule. In the UK there’s now a trend towards portfolio careers, where doctors combine clinical work with other interests – like teaching, research, medico-legal work and media. This, I feel, reduces the risk of ‘burnout’ and promotes the development of new skills. At ‘Infanta Mercedes’, however, doctors work at the practice five days a week – special interests are fulfilled outside working hours.

Nevertheless, the advantage of such an intense working regime is an astonishing continuity of care. Registered patients are *always* seen by their named doctor or nurse. As Camarelles put it, ‘I’ve been looking after the same patients for over nineteen years. I know some of them better than my own family.’

But despite Camarelles’ commitment to the practice, he has little say in how it is run or who works in his team. Spanish GPs are contracted to work directly by the government – a stark contrast to the partnership model of most UK practices.

A particularly valuable lesson was what the exchange taught me about my consultation technique. The paternalistic consultation style, often discouraged in UK training but widely used in Spain, seemed to be very useful when applied appropriately. Despite my previous misconceptions, I did not find it to be patronising, and it appeared to reduce the ‘you tell me, you’re the doctor!’ response I often get when negotiating a management plan. Some Spanish patients do prefer the doctor to choose the best clinical decision on their behalf, thus reducing pressure and anxiety that they will make the ‘wrong’ choice. I have now respectfully incorporated this method into some of my consultations, depending on the patient and their complaint.

Since returning, moreover, I’ve been able to better manage my Spanish patients’ expectations: I now understand why many of them immediately request referrals for child or women’s health, as most GPs in Spain simply don’t deal with these issues. In a city as diverse as London, such lessons can only be a good thing.

Altogether, I highly recommend participating in a *Hippokrates* exchange, either as a visitor or a host. It expanded my knowledge of general practice and widened my circle of international peers. It enabled me to get an insight into another country’s healthcare system. And, perhaps more than anything, it gave me a greater appreciation of my own.

## Note

1. 
http://vdgm.woncaeurope.org/content/about-hippokrates.

